# Electroacupuncture Pretreatment as a Novel Avenue to Protect Brain against Ischemia and Reperfusion Injury

**DOI:** 10.1155/2012/195397

**Published:** 2012-04-26

**Authors:** Xin Li, Peng Luo, Qiang Wang, Lize Xiong

**Affiliations:** ^1^Department of Anesthesiology, Xijing Hospital, Fourth Military Medical University, 127 Changle West Road, Shaanxi, Xi'an 710032, China; ^2^Department of Neurosurgery, Xijing Hospital, Fourth Military Medical University, 127 Changle West Road, Shaanxi, Xi'an 710032, China

## Abstract

Electroacupuncture (EA) pretreatment is a recent observation which has been shown to induce ischemic tolerance mimicking the ischemic pretreatment, suggesting that EA pretreatment may be a promising preventive strategy for the patients with high risk of acute ischemia/reperfusion injury. It was first described in the brain, then in the heart where EA stimulation at acupoint prior to ischemia led to neuroprotection and myocardial protection and induced rapid and delayed ischemic tolerance. Then the optimal parameters and acupoint specificity of EA pretreatment to induce protective effect were proved. Many studies have shown that protective mechanisms of EA pretreatment may involve a series of regulatory molecular pathways including activity enhancement of antioxidant, regulation of the endocannabinoid system, involvement of *beta*-adrenergic receptor, and postreceptor signaling pathway, inhibition of apoptosis. Recently, the neuroprotective and cardioprotective effect of EA pretreatment had been demonstrated in patients undergoing craniocerebral tumor resection or heart valve replacement surgery. Thus, the purpose of this paper is to collect the evidence for the neuroprotective effect of EA pretreatment, to summarize the proposed protective mechanisms of EA pretreatment, and to discuss the possibility of EA pretreatment as a new preventive strategy for patients with high risk of ischemia in clinic.

## 1. Introduction

Stroke is the second most common cause of death and results in a large number of people with disability worldwide. Strokes are either ischemic or hemorrhagic, but more than 80% of stroke cases are caused by cerebral ischemia [1]. Although many neuroprotective agents have been proved to reduce infarction volume and improve neurological recovery in basic research with animal models of stroke, few of them show effects in clinical trials [2, 3]. Moreover, most of the currently used pharmacotherapies are focused on treatment after ischemia, such as tissue plasminogen activator (t-PA), to remove the thrombus and reestablish blood perfusion in ischemic region. Although t-PA facilitates the revascularization when given at the early stage (up to 3 h) after ischemia, the safety and efficacy of t-PA at late stage (beyond 3.5 h) remain controversial [4]. The neuroprotective agent Edaravone, a free radical scavenger, has been approved internationally to treat cerebral ischemia recently. However, it can neither stop the process of infarction or edema after the stroke, nor improve the neurological outcomes of survivals [5]. Although there are some effective drugs like antiplatelets and anticoagulants to prevent stroke, the possibility of causing hemorrhage has limited the application of these drugs. Therefore, it is a huge and urgent medical need to develop novel and rational strategies aimed at preventing ischemia/reperfusion (I/R) injury as well as reducing impairments caused by I/R.

The phenomenon of pretreatment induced ischemic tolerance provides a new idea for the prevention of I/R injury. The pretreatment effect is that a brief exposure to sublethal or noninjurious stimuli can increase resistance to the subsequent prolonged and lethal damage [6]. There are various kinds of pretreatment measures, such as ischemia (regional or remote), hypoxia, endotoxin, cytokines, and anesthetics. Accumulating preclinical evidences has demonstrated that these pretreatment methods, especially ischemia pretreatment, could induce neuroprotection and myocardial protection against I/R injury [7, 8]. But from the clinical point of view, all the mentioned pretreatment ways have limitations and adverse effects to be applied in patients, especially the patients with severe illness.

EA is a novel therapy based on traditional acupuncture combined with modern electrotherapy. Due to the beneficial effects of acupuncture to different brain and heart diseases, EA has been used in treatment as an improvement on traditional acupuncture. Evidence shows that EA not only reduces the myocardial injury but also significantly promotes recovery of neurological function and, thus, improve their quality of life [9].

In recent years, numerous studies have shown that EA also have pretreatment effect, inducing ischemic tolerance as well [10, 11]. Since EA is economical, easily performed, and has few negative side effects, it is clinically applicable for prevention, and not just treatment of ischemic cardiac/cerebral disease. In this paper, we discuss evidence of EA pretreatment induced neuroprotection both from animal experiments and clinical trials, parameters of EA pretreatment, and some mechanisms involved in this effect. Because heart shares lots of characters with brain, especially in I/R injury, and researchers have done lots of work of EA pretreatment on heart, the myocardial protective effects of EA pretreatment are included.

## 2. EA Originates from Acupuncture

Acupuncture, an alternative medicine derived from Chinese traditional medicine, is a procedure in which fine needle is inserted into patients at discrete acupoint and manipulated. Although it is first developed in China, it has spread worldwide and used to treat various diseases [12]. Since stimulation of acupuncture is supposed to be tightly associated with nervous system, acupuncture is expected to improve the neurological function after stroke. Although two systematic reviews indicated that the evidence is not enough to support the positive effect of acupuncture on functional recovery after stroke [13, 14], a lot of clinical trials have verified that acupuncture stimulation improved balance function [15] and spastic states [16], in stroke patients, reduced muscle spasticity and improved motor function for chronic stroke survivors with moderate or severe muscle spasticity [17]. Acupuncture also improved function of the affected upper limb in chronic hemiparetic stroke patients by increasing activity in the ipsilesional motor cortex [18]. In addition, 46% of stroke survivors in the United States engaged in some form of complementary and alternative medicine (CAM) therapy, in which acupuncture was the most frequently used CAM therapy in stroke survivors [19]. However, the beneficial effects of acupuncture in stroke patients required more high-quality evidence [20].

Integrated with electrotherapy, EA is conducted by inserting acupuncture needles into acupoints and then changing electric stimulation parameters, including the stimulation frequency, current intensity, pulse width, and pulse interval [21]. Except for a small current passing through a pair of needles, EA is similar to the regular acupuncture. Thus, EA not only inherits the benefits of traditional acupuncture, but also integrates with the physiological effects of electric stimulation [21].

Compared with regular acupuncture, there are two advantages for EA. On the one hand, the acupoint of EA is not as precise as regular acupuncture, since current delivered by needles stimulates a larger area than that of needle itself. On the other hand, there is an alternative technique for EA, which is called transcutaneous electrical nerve stimulation (TENS). TENS uses electrodes which are taped to the surface of the skin other than needles being inserted, so it can be applied in condition that patients deny insertion of needle or cannot be needled. Based on these two advantages, a growing number of basic researches and clinical trials are preceded in investigating the neuroprotective effect of EA and mechanism of this effect. A large number of animal studies have shown that EA could reduce neural apoptosis, promote cell proliferation, increase cerebral blood flow (CBF), and improve neurological function after stroke [22–25]. These results provide some evidence for further translational studies. So far, some clinical trials have focused on the effects of EA in stroke patients, but the results are ambiguous. Although many investigations with small enrollments support that EA treatment has positive effects on the motor function and quality of life [9, 26], two randomized controlled trials including more patients find that there is no significant difference between EA group and control group in improvement of functional outcome and life satisfaction [27, 28]. In respect to this contradiction, the new method of EA treatment is required for further clinical application.

## 3. “Preventive Acupuncture” and EA Pretreatment

As an indispensable part of traditional Chinese medicine, acupuncture has played an important role in prevention and treatment of diseases throughout history. “Treating before sick” is the plain idea of preventive medicine in traditional Chinese medicine. “Preventive acupuncture” is an approach using acupuncture to “treat before sick.” Namely, applying acupuncture in healthy or mildly sick patients to stimulate the meridians of the body and enhance the body's resistance to disease, in order to prevent disease or to reduce the extent of damage following disease [29]. In general, most of preclinical studies and clinical trials on EA focused on its therapeutic role after stroke. However, prevention is definitely superior to treatment. Since EA is economical, easily operated, and has fewer negative side effects than the other prevention methods (e.g., pharmacological, ischemic, etc.), it should be more valuable and advantageous in preventing ischemic cerebral vascular disease, especially on patients with high risk of ischemic injury.

**Figure 1:**
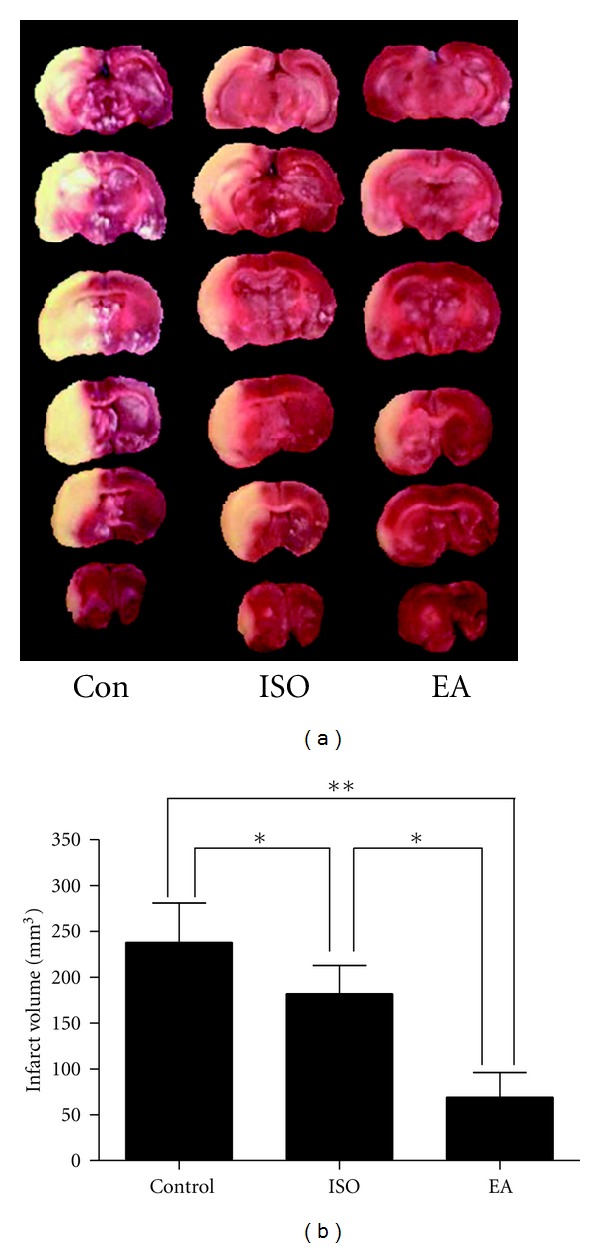
Identification (definition) of EA pretreatment. This graph shows the infarct volume of brain 24 h after MCAO for 120 min in rats. (a) Representative pictures of coronal sections of rat brain after infarction stained with 2,3,5-triphenyltetrazoliumchloride. (b) The bar graph showing the statistical analysis for infarct volumes in 3 groups. CON: control group; ISO: isoflurane pretreatment group; EA: electroacupuncture pretreatment group. (*n* = 10, *P < 0.05, **P < 0.01). This figure was adapted from [10].

In 2003, Xiong and colleagues, the results of whose research were shown in Figure 1, first defined the concept of EA pretreatment. They reported that repeated EA stimulation at the Baihui acupoint (GV20) before cerebral ischemia in rats could significantly reduce infarct volume caused by transient middle cerebral artery occlusion (MCAO) (38.3 ± 25.4 mm^3^ in the EA group versus 220.5 ± 66.0 mm^3^ in the control group and 168.6 ± 57.6 mm^3^ in the anesthetized group) and improve later neurological outcomes [10]. The results showed that EA stimulation before ischemia could produce an effect similar to ischemia pretreatment and induce ischemic tolerance. Later, this group showed that pretreatment with a single EA session could also induce tolerance to focal cerebral ischemia in rats [11]. At the same time, another group [30] found that EA pretreatment at Hegu (LI4), a well-known acupoint commonly used in Oriental medicine for the treatment of neuronal injury resulting from hypoxia-ischemia, could induce neuroprotective effect in neonatal hypoxic-ischemic rat brains.

Just like ischemia pretreatment, EA pretreatment has potential protective effects on mammalian brain. Furthermore, both ischemia pretreatment and EA pretreatment can produce the acute and delayed neuroprotection. A single EA stimulation at Baihui acupoint for 30 minutes could induce biphasic tolerance against focal cerebral ischemia: the acute phase occurred 2 hours after EA pretreatment while the delayed ischemic tolerance was observed 24 hours after the stimulus [11].

The neuroprotective effect of EA pretreatment not only exists in ischemic cerebral injury. Recently, researchers found that EA pretreatment could reduce pathological injury in hippocampal neurons, decrease the expression of activated caspase-3, reduce the number of apoptotic neurons in the CA1 area, and improve learning and memory in rats exposed to high-sustained positive hypergravity (+Gz) [31]. The results showed that EA pretreatment could ameliorate +Gz-induced impairment of learning and memory by inhibiting neuronal apoptosis.

In addition, EA pretreatment was also observed in heart. Researchers applied EA to bilateral Neiguan (PC6) before or during myocardial I/R induced by ligating and reperfusing the left anterior descending coronary artery. The results showed that there were significant reductions in cardiac enzymes, the duration of arrhythmia, and mortality rate in rats that were either preconditioned or treated with EA on PC6, compared with those that did not underwent EA [32].

## 4. Optimal Parameters of EA Pretreatment

Although EA pretreatment could induce neuroprotective effect similar to ischemic pretreatment, there are some characters about EA pretreatment different from other pretreatment ways, especially the electrical stimulation parameters and the acupoint specificity. The parameters and acupoint defining the protective effect of EA pretreatment are summarized in Table 1.

The electric stimulation parameters (frequency, pulse width, current intensity, and duration) and their quantification are important parts of the research on EA. Studies have shown that electric stimulation of different parameters may have different effects on some body functions. Nested design [45] was adopted to identify the influence of different parameters and their combination on EA pretreatment induced cerebral ischemic tolerance in rats. This study at last confirmed the appropriate electrical stimulation parameters of EA pretreatmentto induce cerebral ischemic tolerance in rats: density-sparse wave of 2/15 Hz, current intensity of 1 mA, and 30 min/d for 5 consecutive days. The results showed varying frequency and waveform of the stimulus could produce different protective effects, but there were no significant differences in infarct volume between rats that received stimulation from 1 mA to 3 mA current intensity, indicating that frequency and waveform were probably more important parameters than current. Density-sparse wave had the most obvious neuroprotective effect, followed by intermittent wave, and the continuous wave's neuroprotective effect was relatively poor. The reason for this is probably that continuous wave tended to induce tolerance of the electric stimulus, whereas density-sparse wave could stimulate the release of different types of neurochemicals by transformation between low, medium, and high frequency stimuli. Therefore, the density-sparse wave EA stimulation could generate neuroprotective effect at different targets via the activation of different signaling pathways.

## 5. Acupoint Specificity of EA Pretreatment

Based on meridian theory, an acupoint is relatively specific to certain functions or certain organs, and different effects occur when different acupoints are stimulated. The neuronal specificity of acupoint has been tested by functional magnetic resonance imaging (fMRI), providing neurobiological evidence for the existence of acupoint specificity [46, 47]. Lu et al. found that EA pretreatment of the Baihui acupoint could induce more robust neuroprotection against cerebral I/R injury than stimulation 1 cm lateral to the Baihui acupoint or nonmeridian points of the distal limbs [2]. The Baihui acupoint was chosen because the theory of meridians in traditional Chinese medicine indicates that the Du meridian is closely related to the brain and spinal cord and Baihui is one of the acupoints of the Du meridian. At the same time, according to modern medicine, Baihui acupoint (GV20) is in the projection area of the motor and sensory cortex, as well as in the projection area of the anterior cerebral artery. Therefore, Baihui is probably an important acupoint in preventing and treating cerebral diseases. Similarly, EA pretreatment at Weizhong acupoint (BL40) was more beneficial for spinal cord I/R injury in rabbits than pretreatment at the Tsusanli acupoint (ST36) [48]. Hegu (LI4) was chosen for the treatment of neuronal injury resulting from hypoxia-ischemia [30], and Neiguan (PC6) was preferred in EA pretreatment-induced cardioprotection for its effect on heart disease [32].

## 6. Proposed Protective Mechanisms of EA Pretreatment

**Figure 2:**
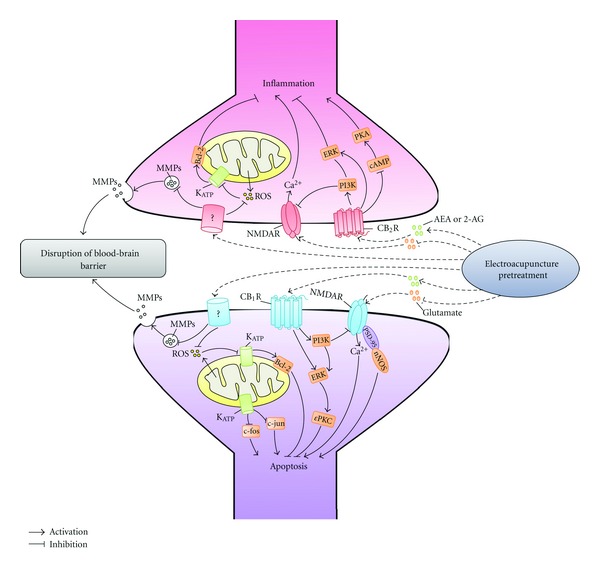
Involvement of cell signaling pathways in neuroprotection of EA pretreatment. This diagram shows the cell signaling pathways involved in the cerebral ischemic tolerance induced by EA pretreatment. PKA: protein kinase A; ERK: extracellular-regulated kinase; cAMP: cyclic adenosine monophosphate; PI3K: phosphatidylinositol 3-kinase; ROS: reactive oxygen species; K_ATP_: ATP-sensitive potassium channel; MMP-9: matrix metalloproteinases-9; NMDAR: N-methyl-d-aspartate receptors; CB2R: cannabinoid receptor type 2; AEA: N-arach-idonoylethanolamine-anandamide; 2-AG: 2-arachidonylglycerol; CB1R: cannabinoid receptor type 1; PSD-95: postsynaptic density 95; nNOS: neuronal nitric oxide synthase; *ε*PKC: epsilon protein kinase C.

EA is pleiotropic and EA pretreatment could have multiple complicated influences on the physiology of brain and on the pathophysiology of cerebral ischemia. Although protective mechanisms of EA pretreatment are largely unknown, a series of studies have shown that EA pretreatment primarily regulates oxidative stress [49], maintains the integrity of blood-brain barrier (BBB) [40], and inhibits apoptosis [30, 33] via different receptors, for example, adenosine receptor type 1 (A1R) [11], opiod receptors [37], cannabinoid receptors (CB1, CB2) [41–44], N-methyl-d-aspartate receptors (NMDARs) [39], and downstream intracellular signaling events including K_ATP_ channels [30], extracellular regulated kinase (ERK) [42], epsilon protein kinase C (*ε*PKC) [44], and phosphatidylinositol 3-kinase (PI3K) pathway [50], while the researches of myocardial protective mechanisms induced by EA pretreatment mainly focus on *β*-adrenoreceptor (*β*-AR) and postreceptor signaling pathway [34–36, 38, 51]. Signaling pathways involved in the neuroprotection of EA pretreatment were summarized in Figure 2.

### 6.1. EA Pretreatment Regulates Oxidative Stress, Maintains the Integrity of BBB, and Inhibits Apoptosis

Abrupt reperfusion after ischemia results in overproduction of reactive oxygen species, which leads to the brain injury [52]. EA pretreatment enhances the activity of mitochondrial respiratory enzymes, attenuates lipid peroxidationand and reduces the production of reactive oxygen species (ROS), consequently improving the function of the respiratory chain and antioxidant capacity in the ischemic penumbra [49, 53]. In addition, it increases the levels of antiapoptotic genes like Bcl-2 while decreasing the levels of proapoptotic genes such as c-Jun and c-Fos, inhibiting subsequent apoptotic cascades [30, 33]. Jiang et al. also found that the antagonizing effect of EA pretreatment on cerebral hypoxic/ischemic injury may be related to its activation of K_ATP_, inhibiting the neuronal apoptosis induced by the immediate genes c-Fos and c-Jun at early injury stages [33].

Moreover, BBB integration and stress reactions are involved in the neuroprotection after EA pretreatment. BBB integration is disrupted by cerebral ischemia, resulting in the brain edema. Matrix metalloproteinases (MMPs) are neutral proteases that disrupt the BBB and are associated with subcortical ischemic vascular disease [54]. The expression and activity of matrix metalloproteinases-9 (MMP-9), one of MMPs, are decreased after EA pretreatment, and subsequently the brain edema and BBB damage are significantly alleviated [40]. This phenomenon has also been observed in another experiment, which indicates that ERK pathway is involved in this process [55].

### 6.2. Endocannabinoid System Contributes to the Neuroprotective Effects of EA Pretreatment

Recent investigations have shown that the endocannabinoid system may be a new mechanism of EA pretreatment-induced neuroprotection. EA pretreatment increases the release of 2-arachidonylglycerol (2-AG) and N-arach-idonoylethanolamine-anandamide (AEA), 2 endocannabinoids and upregulates the expression of cannabinoid CB1 receptor in brain. Selective CB1 antagonist AM251 or CB1 short interfering RNA (siRNA) blocked the neuroprotective effects of EA pretreatment. Meanwhile, pretreatment with 2-AG and AEA also reduced infarct size and improved neurological outcomes [41]. Moreover, further study [43] showed that both acute and delayed ischemic tolerance were associated with endocannabinoid system: the acute phase which occurred in 2 hours after the EA pretreatment was mediated by CB1, whereas the delayed phase occurred in 24 hours after the EA pretreatment via CB2 [43]. These findings indicated that the endocannabinoid system plays an important role in the neuroprotective effect of EA pretreatment.

**Figure 3:**
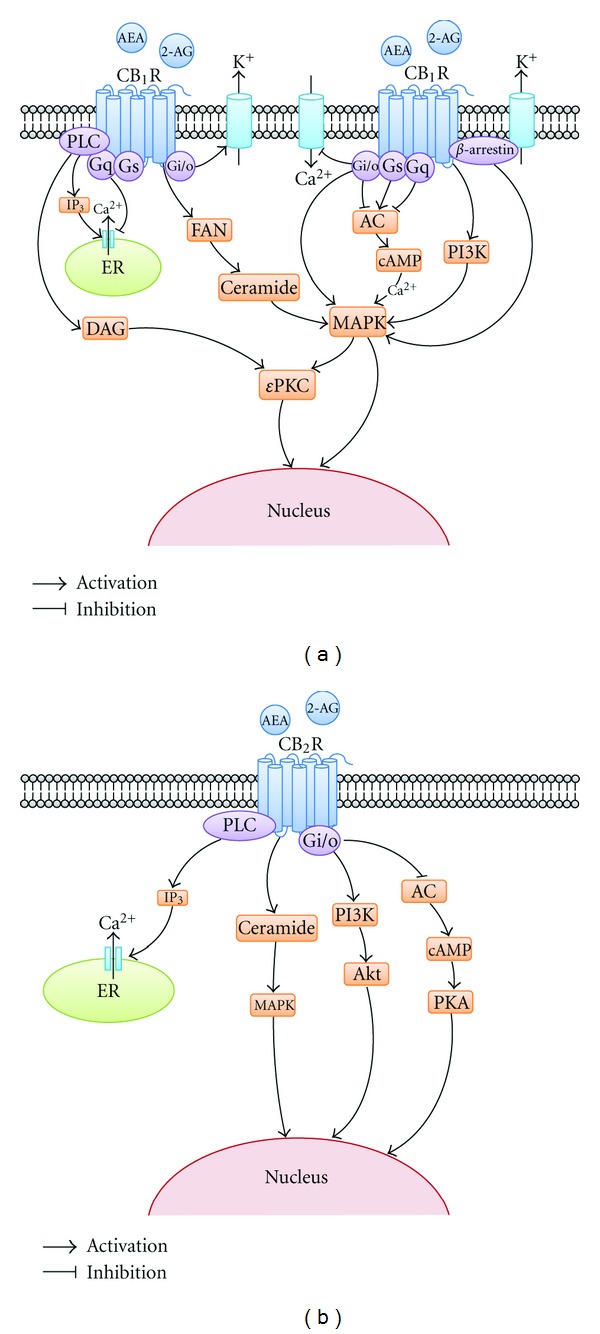
Intracellular signaling pathway of endocannabinoid system in neuroprotection. This graph shows the endocannabinoid system and the post-receptor signaling pathway involved in its neuroprotective effect. (a) signaling pathway of CB1R in neuroprotection; (b) signaling pathway of CB2R in neuroprotection. AEA: N-arach-idonoyl-ethanolamine-anandamide; 2-AG: 2-arachidonylglycerol; CB1R: cannabinoid receptor type 1; PLC: phospholipase c; IP_3_: inositol triphosphate; ER: endoplasmic reticulum; DAG: diacylglycerol; FAN: factor associated with neutral sphingomyelinase; AC: adenylatecyclase; cAMP: cyclic adenosine monophosphate; PI3K: phosphatidylinositol 3-kinase; MAPK: mitogen-activated protein kinase; *ε*PKC: epsilon protein kinase C; PKA: protein kinase A; AKT: protein kinase B.

Activation of the CB1 receptor triggers signaling transduction events that can influence ischemic compensatory responses. Cellular responses that elicit neuroprotection may involve CB1 receptors and their link to a variety of signaling elements, including the Gi/Go family of G-proteins, mitogen-activated protein kinase (MAPK), kinase (MEK1/2), and its substrate, ERK1/2. Further studies have demonstrated that the neuroprotection of EA pretreatment could be abolished by U0126 (a specific inhibitor of the MEK1/2) or TAT-*ε*V1-2 (an *ε*PKC-selective peptide inhibitor). The blockade of CB1 receptor by a CB1 receptor antagonist AM251 reversed the activation of ERK1/2 and *ε*PKC resulted from EA pretreatment. These findings suggest that the ERK1/2 and *ε*PKC pathway might be involved in EA pretreatment-induced cerebral ischemic tolerance via the cannabinoid CB1 receptor [42, 44].  Figure 3 shows the endocannabinoid system and the postreceptor signaling pathway involved in its neuroprotective effect.

### 6.3. EA Pretreatment Attenuates Glutamate Excitotoxicity via NMDAR

Cerebral ischemia induces excessive glutamate release and excitotoxicity [57]. Transient increase of cerebral blood flow (CBF) during reperfusion (hyperemia) would aggravate the brain injury induced by excitotoxicity. Pre-, intra-, or posttreatment of EA could rescue hippocampal neurons from ischemic insults via decreasing the production of glutamate and reducing hyperemia [39, 58]. Previous studies indicated that NMDARs are responsible for glutamate-induced excitotoxicity in the postischemic brain [57, 59]. EA pretreatment suppresses the expression of NR1, a subunit of the NMDARs, which may contribute to its effect in reducing apoptosis and protecting cerebral neurons [39]. Further study suggested that reduced NR1 expression could be reverted by specific inhibitors of the PI3K pathway, but inhibition of the ERK pathway did not show the same effects [50]. Therefore, EA pretreatment attenuates glutamate excitotoxicity by modulating the PI3K pathway.

### 6.4. *β*-Adrenoreceptor and Postreceptor Signaling Pathway Are Involved in Cardioprotection EA Pretreatment

The mechanism of EA pretreatment in the heart is not entirely identical to brain [34–36, 38, 51]. Studies have shown that EA pretreatment can significantly attenuate the incidence of arrhythmia and enhance myocardial cAMP and Gs alpha protein after I/R injury [35, 36]. This attenuating effect was significantly inhibited by the intraperitoneal pretreatment with propranolol, a specific *β*-AR antagonist [34]. These results indicated that EA pretreatment was antiarrhythmic after myocardial I/R, and is mediated by the postreceptor signaling pathway of the *β*-AR. Later, the same group found that adenylatecyclase, protein kinase A, and L-type Ca^2+^ channel, the *β*-AR signaling components modulating intracellular Ca^2+^([Ca^2+^])_i_, were involved in mediating the neuroprotection of EA pretreatment in the isolated rat hearts subjected to simulated global ischemia and reperfusion [38]. Pretreatment with EA can effectively resist myocardial I/R-induced arrhythmia and intracellular calcium oscillation in the rats [51].

## 7. Clinical Application of EA Pretreatment

Although EA pretreatment has been shown to reduce cerebral ischemic injury in numerous preclinical studies, it is unclear whether EA pretreatment could also be applied to clinical practice. The latest clinical trials provide some evidence for the effectiveness of EA pretreatment in patients.

To study neuroprotection after EA pretreatment, Lu et al. enrolled 32 patients requiring selective craniocerebral tumor resection, and randomly assigned them to EA or control groups. At 2 hours before surgery, patients in the EA group received EA stimulation at Fengfu (Du16) and Fengchi acupoint (GB20) for 30 minutes. Patients in the control group received no pretreatment. The results showed that the serum levels of S100 calcium-binding protein *β* (S-100*β*) and neuron-specific enolase (NSE) in the EA group were significantly lower than in the control group at the end of the surgery and 24 hours after surgery [56]. This study indicated that EA pretreatment might have potential protective effects on surgical brain damage.

In the same year, Yang and colleagues also designed a randomized controlled trial enrolling 60 patients to investigate the cardioprotective effect of EA pretreatment in patients undergoing heart valve replacement surgery [60]. EA or sham stimuli were applied at bilateral Neiguan (PC6), Lieque (LU7), and Yunmen (LU2) for 30 minutes each day for five consecutive days before surgery. The level of serum cardiac troponin I was significantly decreased in the EA group at 6, 12, and 24 hours after aortic cross-clamp removal. Meanwhile, EA pretreatment also reduced the inotrope use at 12, 24, and 48 hours after the intensive care unit arrival and shortened intensive care unit stay time [60]. The results demonstrated that EA pretreatment might alleviate cardiac I/R injury in adult patients undergoing heart valve replacements.

**Figure 4:**
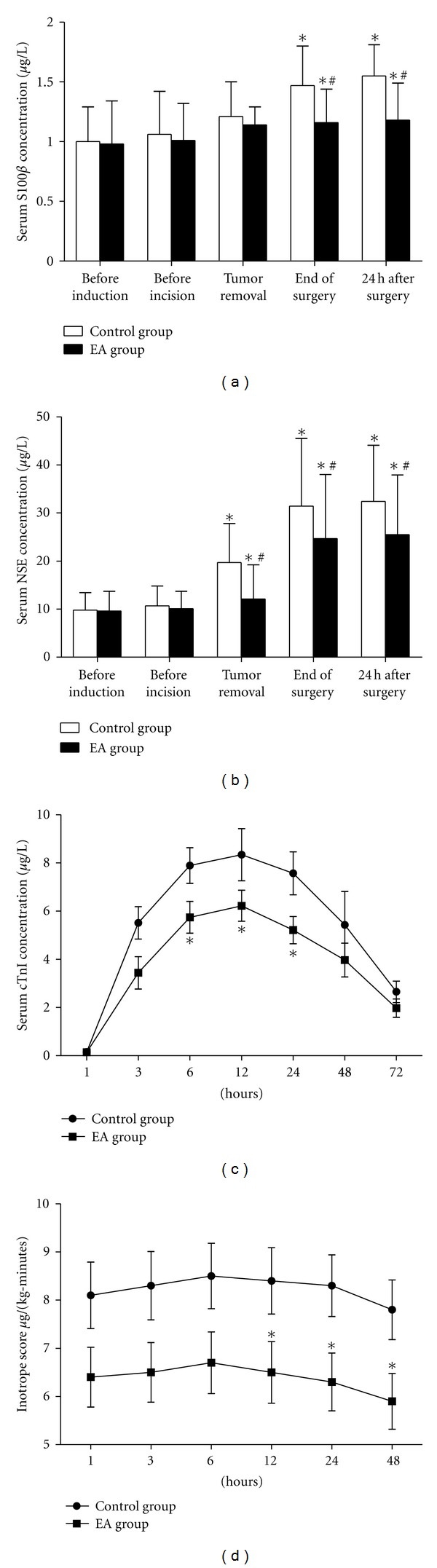
Effects of EA pretreatment in clinical trials. This diagram shows the results of 2 clinical trials concerning the neuroprotection and myocardial protection of EA pretreatment. (a, b) Neuroprotective effect of EA pretreatment in patients undergoing craniocerebral tumor resection ((a) changes of S100*β* at different time points; (b) changes of NSE at different time points). (c, d) Myocardial protection of EA pretreatment in patients undergoing heart valve replacement surgery ((c) serum cTnI levels during 72 hours after the removal of aorta cross-clamping; (d) inotrope scores over the first 48 hours after arrival in the ICU). EA: electroacupuncture; S100*β*: S100 calcium-binding protein *β*; NSE: neuronspecific enolase; cTnI: cardiac troponin I; ICU: intensive care unit. These figures ware adapted from [44, 56].

Results of the two trials were partly showed in Figure 4. These two clinical trials have indicated that EA pretreatment may have beneficial effects on patients undergoing surgery. But, this evidence is limited since the number of enrolled patients is small and both of these trials were conducted in a single center. Thus, multiple center randomized controlled trials are needed to provide further evidence on EA pretreatment. Some other undergoing clinical trials concerning EA pretreatment are summarized in Table 2.

## 8. Conclusion and Prospects of EA Pretreatment

Available data indicates that EA pretreatment can reduce ischemic cerebral injury and improve neurological outcomes. Like other pretreatment methods, EA pretreatment can induce biphasic (acute and delayed) tolerance against cerebral ischemia. However, unlike other pretreatment methods, neuroprotection induced by EA pretreatment is parameter-dependent and acupoint-specific. Multilevel, multipathway, and multitarget mechanisms have been identified in the neuroprotective effect of EA pretreatment.

However, all current studies just provide limited information about mechanisms of EA pretreatment. Just like other pretreatment methods, biochemical, morphological, and behavioral changes induced by I/R injury after EA pretreatment are intensively observed, and EA pretreatment has been proved as the reason for the change. But, little work focuses on the reason why EA treatment before I/R injury can induce change after I/R injury. For example, EA pretreatment has been implicated to induce the overproduction of 2-AG and AEA, thereby activating endocannabinoid system and reducing brain damage and functional deficit after I/R injury. But, it is still unclear why EA treatment before ischemia can induce the change of endocannabinoid system after ischemia. What occurs during the process of EA pretreatment must be explored.

In addition, although compared with other pretreatment methods, EA pretreatment is economical, safe, and easily operated, is more likely to be accepted by patients, and has strong clinical applicability, there is still a gap between potential benefits of EA against ischemia and clinical application of EA in neuroprotection. The benefits of EA pretreatment are tightly associated with determination of EA pretreatment parameters, such as frequency and duration of pretreatment and specific acupoint. In most of current studies, EA pretreatment parameters are selected according to the neuroprotective effects after I/R injury. But, this method is not fit for the clinical application. Therefore, it is necessary to develop preischemic markers indicating the effectiveness of EA pretreatment. Once we find a series of biomarkers for EA pretreatment, it is possible that we would apply individual EA pretreatment due to different genders, age groups, and pathological status such as diabetes. Furthermore, on the basis of basic research and clinical test, there is still not enough data for supporting the neuroprotective effect of EA pretreatment. Thus, multicenter randomized control trials should be carried out and may provide evidence for determining the neuroprotective effect of EA pretreatment and further clinical practice. Although much work remains, if we successfully find satisfying answers to the above problems, we may turn the wishes of patients suffering from cerebral ischemia to live a better life into reality through EA pretreatment.

## Figures and Tables

**Table 1:** Summary of animals experiments on EA pretreatment.

Reference	Species	EA pretreatment		Models	Infarct reduction	Mechanisms
		ESP	Acupoint			
Xiong et al., 2003 [10]	Rats	15 Hz, 1 mA, 30 min/d, 5 d	Baihui (GV20)	2 h MCAO,	~83%, versus control group ~77%, versus isoflurane group	Didnot clarify

Jiang et al., 2004 [33]	Neonatal rats	2/60 Hz, 0.1–0.6-1 mA (10 min each), 30 min	Bilateral Hegu (LI4)	Left pCCAO, 2 h hypoxia	No morphological study	EA pretreatment inhibits the proapoptotic gene via activating K_ATP_

Wang et al., 2005 [11]	Rats	15 Hz, 1 mA, 30 min	Baihui (GV20)	2hMCAO	~55%, EA 2 h before MCAO No protection, EA 0.5, 1, 3 h before MCAO	Rapid ischemic tolerance occurs in 2 h after EA pretreatment, A1R antagonist reverses the neuroprotection

Gao et al., 2006 [34, 35]	Rats	20 Hz, 5 mA, 30 min/d, 3 d	Bilateral Neiguan (PC6)	30 min LADCA ligation, 15 min reperfusion	~47%	Propranolol, antagonist of *β*-AR abolish the cardioprotective effect

Gao et al., 2007 [36]	Rats	20 Hz, 5 mA, 30 min/d, 3 d	Bilateral Neiguan (PC6)	30 min LADCA ligation, 15 min reperfusion	~45%	*β*-AR -Gs-protein-cAMP pathway is involved

Xiong et al., 2007 [37]	Rats	2/15 Hz, 1 mA, 30 min/d, 5 d	Baihui (GV20)	2 h MCAO	~67%	EA pretreatment stimulates the release of enkephalins which bind to *δ*- and *μ*-opioid receptors to induce the neuroprotection

Gao et al., 2008 [38]	Rats	20 Hz, 5 mA, 30 min/d, 3 d	Bilateral Neiguan (PC6)	Isolated heart,SGIR	No morphological study	AC, PKA, and the L-type Ca^2+^channel are involved in the mediation of the antiarrhythmic effect of EA pretreatment

Meng et al., 2008 [39]	Rats	1.7 Hz, 1 mA, 20 min/d, 10 d	Baihui (GV 20), Shenshu (BL 23) Zusanli (ST 36)	1.5 h MCAO	No morphological study	EA pretreatment suppresses the increase of Glu content, downregulates NMDAR 1 mRNA expression in rats brain after I/R injury

Dong et al., 2009 [40]	Rats	15 Hz, 1 mA, 30 min/d, 5 d	Baihui (GV20)	2 h MCAO	~63%	EA pretreatment attenuates brain edema and BBB disruption, decreases MMP-9 expression and activity caused by subsequent cerebral ischemia

Wang et al., 2009 [41]	Rats, Mice	2/15 Hz, 1 mA, 30 min	Baihui (GV20)	2 h MCAO	~38%, 24 h, ~15%, 7days	EA pretreatment activates the endocannabinoid system

Du et al., 2010 [42]	Rats	2/15 Hz, 1 mA, 30 min	Baihui (GV20)	2 h MCAO	~18,24 h	ERK1/2 pathway is involved via CB1

Feng et al., 2010 [31]	Rats	2/15 Hz, 1 mA, 30 min/d, 5 d	Baihui (GV20)	+10 Gz, 5 min,	No morphological study	EA pretreatment attenuates the neuronal apoptosis, preserves neuronal morphology and inhibits the caspase-3 activity, ameliorates the learning and memory function

Ma et al., 2011 [43]	Rats	2/15 Hz, 1 mA, 30 min	Baihui (GV20)	2 h MCAO	~22%, 72 h	CB2 contributed to the delayed neuroprotection, whereas CB1 to the rapid ischemic tolerance

Wang et al., 2011 [44]	Rats	2/15 Hz, 1 mA, 30 min	Baihui (GV20)	2 h MCAO	~21%	EA pretreatment activates endogenous *ε*PKC-mediated antiapoptosis via CB1

The electrical stimulation parameters and acupoints defining the protective effect of EA pretreatment are summarized. The infarct volume reduction induced by EA pretreatment is taken by percentage; most of them were estimated according to the bar graphs, for that the exact number of infarct size was not reported in the cited papers. The potential protective mechanisms discussed in the studies are also summarized. EA: electroacupuncture; ESP: electrical stimulation parameters; MCAO: middle cerebral artery occlusion; pCCAO: permanent common carotid artery occlusion; LADCA: left anterior descending coronary artery; SGIR: simulative global ischemia and reperfusion; K_ATP_: ATP-sensitive potassium channel; A1R: Adenosine A1 receptor; *β*-AR: *β*-adrenoceptors; AC: adenylate cyclase; PKA: protein kinase A; cAMP: cyclic adenosine monophosphate; Glu: Glutamate; NMDAR: N-methyl-d-aspartate receptors; BBB: blood-brain barrier; MMP-9: matrix metalloproteinases-9; ERK1/2: extracellular regulated kinase 1/2; CB1: cannabinoid receptor type 1; CB2: cannabinoid receptor type 2; *ε*PKC: epsilon protein kinase C.

**Table 2:** Ongoing clinical trials on EA pretreatment collected from ClinicalTrials.gov.

ClinicalTrials.gov identifier	Start date	Patient group	Estimated enrollment	Stimulus	Acupoints	Primary outcome measures	Secondary outcome measures
NCT01020266	Dec. 2009	Heart valve replacement surgery	300	5/30 Hz, 0.8–1.9 mA, 30 min/d, 5 d before surgery	Baihui (GV20)	Cerebrovascular complications, Score of neurological defect	S-100*β* and NSE blood level
NCT01020942	Jan. 2010	Elective PCI for coronary stenting	500	2/30 Hz,2–6 mA, 30 min/d, 5 d before surgery	Neiguan (PC 6)	cTnI concentration at 48 hours	Ischemic symptoms, ECG evidence of ischemia, CRP, and MACE at 6 months
NCT01227096	Oct. 2010	Children undergoing repair of CHD	60	After anesthesia induction, prior to surgery	Neiguan (PC 6)	cTnI concentration	Duration of CPB and aortic cross-clamp time, cardiac HFAP and cTnI,8-isoprostane,CRP, cytokines

S-100*β*: S100 calcium-binding protein *β*; NSE: neuron specific enolase; PCI: percutaneous coronary intervention; cTnI: cardiac troponin I; ECG: electrocardiogram; CRP: C-reactive protein; MACE: major adverse cardiac events; CHD: congenital heart defects; CPB: cardiopulmonary bypass; HFAP: heart-type fatty acid-binding protein.
